# Do Biological Maturity and Motor Performance Influence Perceived Player Quality? Evidence from U-15 Soccer Players

**DOI:** 10.3390/sports14080342

**Published:** 2026-08-07

**Authors:** Jakov Marasović, Ante Rađa, Marko Erceg

**Affiliations:** 1 Faculty of Kinesiology, University of Split, 21000 Split, Croatia; jakov.marasovic@kifst.eu (J.M.); ante.rada@kifst.eu (A.R.); 2 Research Group “Bio Banding Classification in Sport”, Faculty of Kinesiology, University of Split, 21000 Split, Croatia

**Keywords:** talent identification, biological maturation, youth football, skill index, quality score

## Abstract

This study had two primary objectives: first, to examine the influence of biological maturity status and motor performance indicators on coach-assessed player quality in U-15 soccer players; and second, to explore differences in estimated biological age between players of different quality levels. A secondary objective was to identify differences in morphological characteristics and motor performance between players belonging to different bio-banded maturity groups. A total of 240 U-15 male players (14.12 ± 0.84 years) were assessed for estimated biological age (Mirwald method), motor performance (speed and agility tests with and without ball), and perceived quality (5-point scale). Ordinal regression showed that biological age (*OR* = 2.47) and slalom performance with and without ball, as well as the 20 m sprint with ball, significantly predicted quality, whereas chronological age had a negative effect (*OR* = 0.44). Biological age differed significantly across quality groups (*F* = 3.65, *p* = 0.007, *η*^2^ = 0.06), remaining significant after controlling for chronological age (*F* = 3.78, *p* = 0.005). Bio-banded groups differed significantly in morphological characteristics and in 20 m sprint performance with and without ball, but most differences were attenuated after adjusting for chronological age. These findings confirm the role of biological maturity in coaches’ perception of player quality, with agility tests appearing less maturity-biased than speed tests, while highlighting ball-oriented performance as a potential predictor of playing success.

## 1. Introduction

Selection and identification processes in younger age categories must consider that players of the same chronological age can differ by up to 5–6 years in their bone age [1,2]. Biological maturation is the main concept that reflects such differences and can be defined in three dimensions: status (the current level of physical development relative to full maturity), time (the age at which specific maturational events occur), and tempo (the rate at which maturation progresses) [3,4]. The greatest impact of biological maturation is recorded from 13 to 15 years of age, precisely during the period when biological heterogeneity within chronological age groups reaches its maximum [5]. Differences in biological maturation cause significant variations in anthropometric characteristics as well as components of motor-functional status, where young players who enter puberty earlier have a significant advantage [3,6,7]. For example, in a recent study, Wenger and Csapo [8] found a connection between biological maturity and linear sprint performance, while Ito et al. [9] identified maturity status as a key determinant of agility performance in youth soccer. Overall, previous research has confirmed that younger players with more advanced maturation are strongly overrepresented in youth soccer, particularly at the regional level, in selection teams, and within national youth squads, where competition for limited places is strongest [10,11,12,13,14].

Silvino et al. [15], in their scoping review, found that speed (52.1%) and biological maturity (42.1%) were the most frequently used indicators when identifying talent in youth soccer, followed by lower extremity strength (40.5%), aerobic capacity (35.8%) and agility (32.6%), while technical (16.8%) and tactical skills (14.2%) were represented to a much lesser extent. A special emphasis can be placed on biological maturity, given the possible interindividual differences in the degree of physical development, which raises the question of its influence on the assessed player performance. Hill et al. [11], in a longitudinal study of 278 male players rated on a four-point scale, highlight the consistently better ratings of players after the accelerated growth and development phase (APHV). Furthermore, Hill et al. [12], in a subsequent longitudinal study, confirmed that biological age is a significant positive predictor of coach performance ratings, especially in the U-10, U-14, and U-15 age groups. Moreover, Hill et al. [13], using a longitudinal mixed-effects model on a sample of 98 players aged 12 to 16, and in interviews with nine coaches, found that the status and timing of biological maturity had significant implications for player selection and release decisions, even among coaches who were aware of the issue. Such a systemic pattern can lead to young players who are developmentally delayed being systematically excluded before their capacities are fully realized, which Cumming et al. [16] refer to in practice as the “central mechanism of maturity bias in academy soccer.” Paradoxically, research by Zuber et al. [17] showed that late-maturing players who “survived” the selection process possessed superior technical skills and more adaptive psychological characteristics than their early-maturing peers. Bio-banding has emerged as a practical strategy to address the challenges arising from unequal biological development in youth athletes. By grouping players according to their maturity status rather than chronological age, this approach reduces the variability in maturity-dependent parameters within a given age group, creating a fairer competitive environment in which players compete against their “biological peers.” Neutralizing the physical advantages of more mature players allows greater attention to be directed toward quality parameters that are largely independent of biological maturity, such as technical, tactical, and cognitive skills [18,19].

Despite the growing body of literature in recent years, a holistic approach to the selection and identification of young football talent remains essential. Biological maturity can be regarded as a predictor of competitive performance and a criterion for differentiating players of varying competitive success. Integrating indicators of motor performance as additional factors that may influence assessed quality allows for a more comprehensive evaluation, while the introduction of bio-banding groupings can further clarify the extent of inter-individual differences within the same age category. This study had two primary objectives: first, to examine the influence of biological maturity status and motor performance indicators on coach-assessed player quality in U-15 soccer players; and second, to explore differences in estimated biological age between players of different quality levels. A secondary objective was to identify differences in morphological characteristics and motor performance between players belonging to different bio-banded maturity groups.

## 2. Materials and Methods

### 2.1. Participants

This was a cross-sectional, observational study, in which biological maturity status, motor performance, and coach-rated player quality were assessed at a single time point, without any experimental manipulation of training or competitive exposure. The sample for this cross-sectional study consisted of 240 young male soccer players with a chronological age of 14.12 ± 0.84 years and an estimated biological age of 14.13 ± 1.08 years (body height: 169.35 ± 9.93 cm; body mass: 55.64 ± 10.47 kg; BMI: 19.23 ± 2.10 kg/m^2^ for the overall sample), recruited from clubs competing at various levels of the national league competition. Participants were recruited through a purposive sampling procedure, in cooperation with club coaches and technical staff, from clubs that agreed to participate in the study. All male players registered in the U-15 age category at the participating clubs were eligible for inclusion. Players were included if they were actively engaged in regular team training and competition at the time of testing and had no musculoskeletal injury or illness in the four weeks preceding testing that could affect motor performance. Players with incomplete anthropometric or performance data were excluded from the corresponding analyses. The subjects trained four times per week, while official matches were played on weekends, with an average of about 30 matches per year. All measurements were conducted under standardized conditions (8:00–10:00 AM; 25.6 ± 0.8 °C; 36.3 ± 2.5% relative humidity). At the start of testing, participants completed a questionnaire that included date of birth. Written informed consent was obtained from each parent or legal guardian. All procedures were conducted in accordance with the ethical standards of the institution responsible for the research and the principles outlined in the Declaration of Helsinki, and the study was approved by the Ethics Committee of the Faculty of Kinesiology (approval number 2181-205-02-05-26-026, Split, Croatia).

### 2.2. Procedures

#### 2.2.1. Anthropometric Measurements and Biological Age Assessment

In this study, standardized anthropometric procedures were used to measure the following variables: body height (BH; cm), body mass (BM; kg), seated height (SH; cm), and body mass index (BMI; kg/m^2^). Body height and seated height were measured to the nearest 0.1 cm using a portable stadiometer (seca 213, Seca GmbH & Co. KG, Hamburg, Germany). with the participant standing barefoot in the Frankfurt plane, and body mass was measured to the nearest 0.1 kg using a calibrated digital scale (Seca 876, Seca GmbH & Co. KG, Hamburg, Germany), with participants wearing light athletic clothing and no footwear; each measurement was taken once by a trained member of the research team following standardized anthropometric procedures. Estimated biological age was derived using the sex-specific maturity offset equation of Mirwald et al. [20], which predicts the time (in years) before or after the attainment of peak height velocity (PHV) from chronological age, BH, BM, sitting height, and leg length. This non-invasive, regression-based approach has been widely validated and adopted as the standard method for estimating somatic maturity status in youth athletic populations [3]. For boys, the maturity offset (in years) was calculated as: Maturity offset = −9.236 + (0.0002708 × (leg length × sitting height)) + (−0.001663 × (chronological age × leg length)) + (0.007216 × (chronological age × sitting height)) + (0.02292 × ((body mass/body height) × 100)), where leg length was calculated as body height minus sitting height, and the body mass/body height ratio was expressed as body mass (kg) divided by body height (cm) [20]. Age at peak height velocity (APHV) was calculated by subtracting the predicted maturity offset from chronological age, and estimated biological age was computed as chronological age plus the difference between a sex-specific reference age at PHV (13.8 years for boys) and the individual’s predicted APHV.

#### 2.2.2. Quality Score Classification (QS)

Player quality was operationalized using a composite quality score (QS) on a scale from 1 (poorest) to 5 (best), derived from a two-stage categorization procedure that combined subjective coach ratings with the player’s club competitive level [21]. First, each player’s head coach rated the player’s overall quality on a three-point scale (1 = below average, 2 = average, 3 = above average), based on a standardized assessment of the player’s technical, tactical, and overall performance relative to same-age peers. All rating coaches held at least a UEFA B coaching license and at least 3 years of experience coaching players in the U-13–U-15 age categories, and each player was evaluated by his own head coach based on familiarity with his performance over the preceding competitive season. Coaches were not provided with players’ anthropometric measurements or estimated biological age at the time of rating, reducing the risk that ratings were directly biased by physical appearance. Formal inter-rater reliability could not be assessed, as each player received a single rating from his own head coach rather than from multiple independent raters. Second, this coach-rated score was adjusted for the player’s club’s competitive level [21]. For players from elite clubs (*n* = 89, 37.1%), competing in the first national league, the coach-rated score was shifted to the upper end of the 5-point scale (i.e., a coach rating of 1, 2, or 3 was coded as 3, 4, or 5). Subsequently, players’ quality from regional-level clubs (*n* = 64, 26.7%) was coded as 2, 3, or 4, while players from county leagues (*n* = 87, 36.2%) retained scores of 1, 2, or 3. Additionally, players who were members of the national team were automatically assigned a score of 5, regardless of their club’s competitive level.

#### 2.2.3. Bio-Banding Classification

Following the calculation of estimated biological age for each participant, the sample mean was computed for the U-15 group. Participants were then classified into three maturity groups based on their deviation from the group mean: less mature (more than 0.5 years below the mean), average mature (within ±0.5 years of the mean), and more mature (more than 0.5 years above the mean). This approach of grouping players by maturity status relative to a central reference value is consistent with previous research [22]. To verify that this maturity-based selection concern was not driving the QS classification itself, we cross-tabulated the three competitive tiers against the bio-banded maturity groups: the distribution of maturity groups did not differ significantly across tiers (χ^2^ = 6.77, *p* = 0.148), indicating that more mature players were not disproportionately concentrated in higher-tier clubs in this sample.

#### 2.2.4. Motor Performance Testing

The non-ball performance included tests to assess speed and agility with and without the ball, with results expressed in seconds (s). Sprint ability was assessed using 20-m tests (S20 m), based on standard indicators of acceleration and maximal speed capacity in young soccer players [23]. Agility was assessed using the slalom test (SLAL). The slalom test consisted of moving between six cones placed in a straight line, 2 m apart, for a total length of 11 m. The subject’s task was to complete the designated course as quickly as possible in both directions, with a turn around the last cone. The ball tests included a 20-m sprint (S20 m B) and a ball slalom test (SLAL B), following an approach that combines linear sprinting, agility, and dribbling to assess soccer-specific motor skills. All timing measurements were conducted using a photoelectric cell system (Brower Timing Systems, Draper, UT, USA), whose validity and reliability have been previously established in the sports science literature, with a reported accuracy of 0.01 s [24]. Participants started 50 cm behind the starting line to avoid premature activation of the measuring sensors.

To quantify the technical performance of ball control, two skill index (SI) indicators were calculated as the difference between the results of the same test with and without the ball: sprint skill index (S20 SI = S20 m B − S20 m) and slalom skill index (SLAL SI = SLAL B − SLAL), following the approach previously used by Rađa [21] to isolate ball-handling skill from underlying physical capacity in young soccer players. Higher SI values indicate a greater drop in performance (i.e., a more time lost when dribbling with the ball, which reflects a relatively weaker level of ball control in relation to the subject’s physical capacity).

### 2.3. Statistical Analysis

Descriptive statistics (M, mean; SD, standard deviation; and *n*, sample size) were calculated for all variables, both for the overall sample and separately for each group. The normality of the distribution of all continuous variables was assessed using the Shapiro–Wilk test; given the sample size (*n* = 240), statistically significant deviations from normality were detected for several variables, which motivated the use of robust and nonparametric post hoc procedures (Games–Howell test, Welch’s *t*-test) described below rather than assuming normality throughout. Differences between groups were analyzed using one-way analysis of variance (ANOVA), while analysis of covariance (ANCOVA) was used to examine group differences with chronological age included as a covariate, yielding adjusted means and standard errors of the adjusted means (*M* ± *SE*). Before conducting the ANCOVA, the assumption of homogeneity of regression slopes (group × covariate interaction) was tested and was met for all analyzed variables (*p* > 0.05). Effect sizes were calculated using eta-squared (*η*^2^) for ANOVA and partial eta-squared for ANCOVA, and interpreted according to Cohen’s [25] criteria (small ≥ 0.01, medium ≥ 0.06, large ≥ 0.14). Post hoc comparisons for the ANCOVA-adjusted means were conducted using Bonferroni-corrected pairwise *t*-tests based on the pooled model residual variance. Post hoc comparisons for the quality-based groups (raw values) were conducted using the Games–Howell test, given the unequal group sizes and heterogeneity of variance [26]. Post hoc comparisons for the biological maturity groups (raw values) were conducted using Welch’s *t*-test, which is robust to unequal variances [27]. Effect sizes for all post hoc comparisons (raw and ANCOVA) were quantified using Cohen’s d (*d*) and interpreted according to Cohen [25] (trivial < 0.20, small 0.20–0.49, medium 0.50–0.79, large ≥ 0.80); for the ANCOVA comparisons, d was calculated as the ratio of the difference in adjusted means to the model’s residual standard deviation. A two-tailed significance criterion of α = 0.05 was applied throughout. To examine the joint contribution of estimated biological age, chronological age, and motor performance indicators with and without the ball to the player quality category (QS), an ordinal logistic regression based on a proportional odds model was conducted, with estimated biological age and chronological age entered as separate predictors. All continuous predictors were standardized (z-scores) before entry. Given the correlation between estimated biological age and chronological age, variance inflation factors (VIF) were computed for all predictors to assess multicollinearity. Variance inflation factors (VIF) for all predictors in the model were below 5 (estimated biological age: VIF = 3.43; chronological age: VIF = 3.54; motor performance variables: VIF = 1.83–4.29). The association between competitive tier and bio-banded maturity group was tested using a chi-square test of independence. Overall model fit was assessed using the χ^2^ likelihood ratio test against the null model, and the magnitude of the explained variance was expressed using the McFadden pseudo-R^2^. The effects of individual predictors were expressed as odds ratios (*OR*) with corresponding 95% confidence intervals. Data were processed using IBM SPSS Statistics (version 29.0, IBM Corp., Armonk, NY, USA).

## 3. Results

Table 1 shows the differences in estimated biological age between groups of different quality. Significant differences were found between groups (*F* = 3.654, *p* = 0.007, *η*^2^ = 0.059). Using the Games–Howell post hoc test, significant differences were found between Q1 vs. Q4 (*t* = 2.872, *p* = 0.006, *d* = 0.682), Q1 vs. Q5 (*t* = 2.815, *p* = 0.007, *d* = 0.767), Q3 vs. Q4 (*t* = 2.471, *p* = 0.015, *d* = 0.423), and Q3 vs. Q5 (*t* = 2.350, *p* = 0.022, *d* = 0.497). When chronological age was entered as a covariate, the model remained significant (*F* = 3.778, *p* = 0.005, *η*^2^ = 0.061), with the only significant pairwise difference remaining between Q3 and Q5 (*t* = 3.605, *p* = 0.004, *d* = 0.760).

Table 2 presents the results of the ordinal logistic regression (proportional odds model) predicting the quality category of U-15 players based on estimated biological age, chronological age, and motor performance tests with and without the ball. The overall model was significant (χ^2^ (6) = 120.38, *p* < 0.001) and explained a substantial portion of the variance in player quality ratings (McFadden R^2^ = 0.165). Estimated biological age and the 20-m sprint and slalom tests performed with the ball were significant predictors of quality, in the directions reported in Table 2, whereas chronological age had a significant negative effect and the 20-m sprint without the ball was not a significant predictor.

As a sensitivity check for the collinearity between estimated biological age and chronological age reported above, the model was re-estimated using the maturity offset as a single predictor; this predictor remained significant (*B* = 0.51, *OR* = 1.66, 95% CI 1.29–2.15, *p* < 0.001), with model fit comparable to the original specification (Table S1).

Table 3 shows differences in quality, anthropometric characteristics, and motor performance indicators between bio-banded groups of players. Because the bio-banded groups were themselves defined according to each player’s deviation from the sample mean estimated biological age (Section 2.2.3), the very large effect observed for estimated biological age in Table 3 reflects the grouping procedure itself and should be interpreted as a manipulation check, not a substantive result. Significant differences were found in the following anthropometric characteristics: BH (*F* = 168.527, *p* < 0.001, *η*^2^ = 0.587), with differences among all three groups: Less mature vs. Average (*t* = 10.905, *p* < 0.001, *d* = 1.761), Less mature vs. More mature (*t* = 17.597, *p* < 0.001, *d* = 2.592), and Average vs. More mature (*t* = 5.626, *p* < 0.001, *d* = 0.935); BM (*F* = 160.068, *p* < 0.001, *η*^2^ = 0.575), also with differences among all groups: Less mature vs. Average (*t* = 10.783, *p* < 0.001, *d* = 1.831), Less mature vs. More mature (*t* = 17.443, *p* < 0.001, *d* = 2.575), and Average vs. More mature (*t* = 4.917, *p* < 0.001, *d* = 0.817); and in BMI (*F* = 43.529, *p* < 0.001, *η*^2^ = 0.269): Less mature vs. Average (*t* = 6.170, *p* < 0.001, *d* = 1.036), Less mature vs. More mature (*t* = 8.917, *p* < 0.001, *d* = 1.316), and Average vs. More mature (*t* = 1.994, *p* = 0.048, *d* = 0.330). Significant differences were also confirmed in chronological age (*F* = 170.418, *p* < 0.001, *η*^2^ = 0.590), with all three groups differing from one another: Less mature vs. Average (*t* = 7.617, *p* < 0.001, *d* = 1.312), Less mature vs. More mature (*t* = 19.112, *p* < 0.001, *d* = 2.814), and Average vs. More mature (*t* = 7.647, *p* < 0.001, *d* = 1.360); in the APHV indicator (*F* = 58.526, *p* < 0.001, *η*^2^ = 0.331): Less mature vs. Average (*t* = 4.400, *p* < 0.001, *d* = 0.752), Less mature vs. More mature (*t* = 11.178, *p* < 0.001, *d* = 1.645), and Average vs. More mature (*t* = 4.585, *p* < 0.001, *d* = 0.819); and in estimated biological age, where the largest effect size was recorded (*F* = 653.429, *p* < 0.001, *η*^2^ = 0.846), with all three groups differing significantly (Less mature vs. Average *t* = 18.418, Less mature vs. More mature *t* = 33.814, Average vs. More mature *t* = 17.627, all *p* < 0.001). Quality (QS) showed a statistically significant difference between groups (*F* = 3.261, *p* = 0.040, *η*^2^ = 0.027), with a significant difference only between the Less mature and More mature groups (*t* = 2.637, *p* = 0.009, *d* = 0.389). Regarding off-ball motor performance, S20 significantly differentiated the groups (*F* = 39.294, *p* < 0.001, *η*^2^ = 0.249), with differences among all three: Less mature vs. Average (*t* = 4.775, *p* < 0.001, *d* = 0.861), Less mature vs. More mature (*t* = 9.305, *p* < 0.001, *d* = 1.373), and Average vs. More mature (*t* = 2.147, *p* = 0.034, *d* = 0.384); the slalom without the ball (SLAL) was not a significant discriminator (*F* = 0.982, *p* = 0.376, *η*^2^ = 0.008). Regarding the ball-handling indicators, S20 B showed a significant difference (*F* = 24.033, *p* < 0.001, *η*^2^ = 0.169), with differences among all groups: Less mature vs. Average (*t* = 3.585, *p* < 0.001, *d* = 0.605), Less mature vs. More mature (*t* = 7.013, *p* < 0.001, *d* = 1.032), and Average vs. More mature (*t* = 2.179, *p* = 0.032, *d* = 0.386); whereas SLAL B did not reach significance (*F* = 0.627, *p* = 0.535, *η*^2^ = 0.005). The skill indices did not show significance at the main effect level (S20 SI: *F* = 0.437, *p* = 0.647, *η*^2^ = 0.004; SLAL SI: *F* = 1.112, *p* = 0.331, *η*^2^ = 0.009).

Table 4 presents the differences between bio-banded groups of players after adjusting for chronological age (ANCOVA). The morphological and maturity characteristics remained significant with large effect sizes. Significant differences were confirmed in BH (*F* = 67.191, *p* < 0.001, *η*^2^ = 0.363), with differences among all three groups: Less mature vs. Average (*t* = 10.289, *p* < 0.001, *d* = 1.740), Less mature vs. More mature (*t* = 17.525, *p* < 0.001, *d* = 2.584), and Average vs. More mature (*t* = 4.968, *p* < 0.001, *d* = 0.844); in BM (*F* = 64.208, *p* < 0.001, *η*^2^ = 0.352): Less mature vs. Average (*t* = 10.153, *p* < 0.001, *d* = 1.717), Less mature vs. More mature (*t* = 17.077, *p* < 0.001, *d* = 2.518), and Average vs. More mature (*t* = 4.715, *p* < 0.001, *d* = 0.801); and in BMI (*F* = 17.723, *p* < 0.001, *η*^2^ = 0.131): Less mature vs. Average (*t* = 5.740, *p* < 0.001, *d* = 0.971) and Less mature vs. More mature (*t* = 8.665, *p* < 0.001, *d* = 1.278). The APHV indicator (*F* = 190.692, *p* < 0.001, *η*^2^ = 0.618) retained a large effect, with differences among all three groups: Less mature vs. Average (*t* = 13.432, *p* < 0.001, *d* = 2.272), Less mature vs. More mature (*t* = 30.492, *p* < 0.001, *d* = 4.496), and Average vs. More mature (*t* = 13.095, *p* < 0.001, *d* = 2.224); the same was observed for estimated biological age (*F* = 197.562, *p* < 0.001, *η*^2^ = 0.626): Less mature vs. Average (*t* = 13.671, *p* < 0.001, *d* = 2.312), Less mature vs. More mature (*t* = 31.036, *p* < 0.001, *d* = 4.576), and Average vs. More mature (*t* = 13.330, *p* < 0.001, *d* = 2.264). In contrast, most motor performance differences were substantially attenuated after controlling for chronological age. The 20-m sprint (S20) dropped to a small effect (*F* = 3.699, *p* = 0.026, *η*^2^ = 0.030), with the Less mature group differing from the Average (*t* = 2.943, *p* = 0.011, *d* = 0.498) and More mature (*t* = 3.448, *p* = 0.002, *d* = 0.508) groups. The slalom without the ball (SLAL) became significant with a small effect (*F* = 5.679, *p* = 0.004, *η*^2^ = 0.046), with the Less mature group differing from the Average (*t* = 3.321, *p* = 0.003, *d* = 0.562) and More mature (*t* = 4.831, *p* < 0.001, *d* = 0.712) groups. The 20-m sprint with the ball (S20 B) lost significance (*F* = 1.320, *p* = 0.269, *η*^2^ = 0.011). The slalom with the ball (SLAL B; *F* = 2.850, *p* = 0.060, *η*^2^ = 0.024) retained only a difference between the Less mature and More mature groups (*t* = 3.715, *p* < 0.001, *d* = 0.548). Quality (QS) was no longer significant after adjustment (*F* = 0.358, *p* = 0.699, *η*^2^ = 0.003), nor were the skill indices (S20 SI: *F* = 0.488, *p* = 0.614, *η*^2^ = 0.004; SLAL SI: *F* = 1.187, *p* = 0.307, *η*^2^ = 0.010), indicating that these indicators are independent of biological maturity once chronological age is accounted for.

## 4. Discussion

This study had two primary objectives: first, to examine the influence of biological maturity status and motor performance indicators on coach-assessed player quality in U-15 soccer players; and second, to explore differences in estimated biological age between players of different quality levels. A secondary objective was to identify differences in morphological characteristics and motor performance between players belonging to different bio-banded maturity groups. Age (both biological and chronological), together with the motor variables assessing agility with and without the ball and speed with the ball, showed a significant effect on estimated quality. In addition, differences in estimated biological age between quality groups were found both before and after controlling for chronological age. Finally, significant differences favoring the more mature players emerged in most of the morphological and motor variables examined, although these effects were attenuated when chronological age was included as a covariate.

There are an increasing number of studies on the parameters that influence selection processes in soccer, and most of these studies [13,28] focus on a binary comparison (elite and non-elite groups). Observing the results of this study and comparing them with the results of previous studies, a universal phenomenon can be observed when considering biological maturity in the context of a variable that can differentiate players of different qualities. For example, Aquino et al. [29], in a sample of young soccer players (*n* = 66, mean age = 16.18 ± 0.63 years), found that selected players had a greater maturity offset (1.90 ± 0.59 vs. 1.31 ± 0.40 years) than non-selected players, indicating more advanced maturity among the selected group. Although the present study expressed maturity as estimated biological age rather than maturity offset, both metrics are derived from the same predictive equation and reflect the same underlying construct. Our results extend this insight, showing that the advantage of more advanced maturity is not confined to binary selection but extends across all levels of perceived quality within the entire sample (Table 1). A comparable pattern was reported by Sweeney et al. [14], who found that maturity bias intensifies as competition level rises, with a 0.36 standard deviation increase in body mass, an indirect marker of biological maturity, between regional and international squads. Whereas Sweeney et al. examined body mass across competition levels, our study assessed estimated biological age across quality levels; despite these differing indicators, both findings point to the same underlying tendency, namely that progression toward higher performance levels is accompanied by more advanced biological maturity. In our sample, this was most evident between the Q1 and Q5 groups, which differed by 0.80 years in estimated biological age. This trend can mask other parameters as possible criteria (technical and tactical) that may not be dependent on the current growth and development status. Similar results were also confirmed by Konarski et al. [30] who, in their study on a sample of 31 U-15 players divided into two groups by quality level (select vs. non-select), determined that there are differences in estimated biological age in favor of the select players (15.1 vs. 14.3). Furthermore, some studies have controlled for chronological age when comparing physical characteristics between selected groups [30,31] or included relative age [10,32] as a mediator of maturational differences. Interestingly, after introducing the covariate of chronological age, although the main effect remained significant, the differences in estimated biological age were substantially reduced to only one pairwise difference (Q3 vs. Q5). Of particular interest, the QS groups did not show a statistically significant difference in chronological age (*F* = 2.306, *p* = 0.059), which suggests that the reduction in group differences is not a result of chronological heterogeneity in the sample, but rather of high correlation between chronological and estimated biological age at the individual level (*r* = 0.83). This finding is consistent with research by Malina et al. [33], who report that correlations between predicted maturity offset and chronological age within a single age group were moderately to highly positive (*r* = 0.64 to *r* = 0.87), confirming the thesis that chronological age can explain part of the variance in estimated biological age independently of the absence of group differences in chronological age.

The results of this study showed that all three bio-banded groups differed in anthropometric indicators (large effect), which is consistent with previous studies [34,35,36]. Regarding motor performance, the 20-m sprint test was the most discriminative among the observed maturity groups (large effect). These findings are consistent with the research of Lehnert et al. [37], who, in a sample of 98 young players, found differences between maturity categories in maximal speed tests over a 20–30 m segment (*p* = 0.024). Similar findings were confirmed by Fernández-Galván et al. [38], who determined that longer sprints involving maximal speed (*ES* = 1.54–1.92) are considerably more susceptible to the influence of biological maturity. Regarding agility (slalom test), no significance was found in differentiating groups of different biological maturity (trivial effect). These findings are consistent with the research by Giuriato et al. [39], who, in a sample of 39 young soccer players (13.56 ± 0.58 years), showed that the agility test (Y-Agility Test) does not reveal significant differences between maturation groups (early-average mature: *p* = 0.450; early-late mature: *p* = 1.000; average-late mature: *p* = 0.830). Furthermore, Ito et al. [9] reported a significant increase in the agility test score (Reactive Shuttle Test) between the Pre-PHV and Circa-PHV phases (*d* = −2.75), whereas in the Post-PHV phase progress stalled despite continued physical maturation. Such findings suggest that agility, in its component, includes cognitive factors that play a more significant role than previously thought and that these do not necessarily develop in parallel with physical maturity [39]. Regarding ball-handling performance, significant differences were found in the 20-m sprint test (large effect), while the slalom test (trivial effect) did not prove to be a significant discriminator between the developmental maturity groups. Generally speaking, research comparing ball-handling performance between maturational categories has generally not found significant differences, suggesting that technical skills depend more on training and experience than on biological maturity [40,41,42]. For example, Huijgen et al. [43] in their longitudinal study found that sprinting progresses more gradually in the early stages of development, while significant changes in ball dribbling can only be observed after the age of 16.

In this study, the Skill Index measure was also used as a quantitative indicator of technical skill because it reflects how well a player maintains performance under on- and off-ball conditions [44,45]. Since biological maturity largely determines fundamental motor capacities, comparing the SI between maturity groups allows for distinguishing those players who achieve their better results solely due to greater physical maturity from those who have more successfully mastered the ball control technique [46]. This study did not find significant differences in the two Skill Index measures (S20 SI and SLAL SI), which may suggest that biological maturity has a limited role in the variance of this indicator and that other factors better explain the results. The results of this study confirm that more advanced biological maturity is primarily manifested through anthropometric differences, while its influence on other variables weakens when chronological age is included as a covariate. Although previous studies have used different methodological and statistical approaches when including the covariate of chronological age [47,48], the fact is that chronological age plays a large role in explaining the differences, given that the significant difference in chronological age between the more mature and less mature groups in the study averaged 1.47 years (a large effect). These results further justify the methodological approach, stating that without controlling for chronological age, some of the differences, primarily in motor indicators, between the mature groups may reflect this chronological difference. Summarizing the findings for the tests assessing speed and agility with and without the ball, it can be seen that the discriminative power of the speed tests in differentiating between maturity categories is greater than that of the agility tests. The slalom test was especially telling: the raw values showed no differences between maturity groups (*p* = 0.376), but a significant effect favoring the less mature group appeared once chronological age was controlled (*p* = 0.004). This is likely because agility depends mainly on coordination and technique rather than on the size and strength gained through maturation, so the similar raw performance of the more mature players reflected their older age rather than their maturity. This may serve as a potential “performance indicator” for coaches that is independent of the effects and advantages conferred by more advanced biological maturity.

The results of the ordinal logistic regression showed that estimated biological age was the only significant positive predictor of quality. These results are consistent with the research by Hill et al. [12], who applied multilevel modeling to a sample of 279 players (U-9–U-16) in an English professional academy to predict coach ratings on a 4-point Likert scale. Their results showed that chronological age significantly predicts coach ratings in the U-10, U-14, and U-15 age groups, with more advanced, mature players receiving higher ratings. Also, Hill et al. [11] confirmed that coach ratings decline during the growth spurt phase and rise again in the post-growth spurt phase at U-15, emphasizing that coaching evaluations vary according to the stage of maturity and growth rate. An interesting finding of this study is the negative effect of chronological age on perceived quality. When both age variables are included in the model, biological age takes over in explaining the player’s actual maturity. In contrast, chronological age captures developmental status; a chronologically younger player who has reached the same biological maturity as older teammates is perceived as higher quality because it suggests greater potential for further development. In contrast, an older player at the same level of maturity suggests they are closer to their developmental maximum. This coaching logic is consistent with the qualitative findings of Hill et al. [13], according to which, coaches, when evaluating potential, consider a player’s room for further physical development, not just their current performance level.

Regarding the motor variables that influenced the coaches’ perceived quality, the agility variables (SLAL and SLAL B) and the sprint-with-ball variable (S20 B) stood out as significant predictors. This is in line with Höner et al. [49], who, using logistic regression on a sample of 13,869 players (U-12–U-15), showed that sprinting, dribbling, and tactical skills predict success in professional academies (Nagelkerke R^2^ = 0.15–0.20). Although our study did not find a significant effect of linear sprinting without the ball (S20), a parallel can be drawn with the S20 B and SLAL B variables, which integrates the sprinting and ball-control elements assessed in that study and which emerged as a significant predictor. Such results, where agility is the dominant predictor of quality, are also confirmed by Menezes et al. [50], who, in a sample of 87 U-15 players, even after controlling maturity status, found that agility remains a key factor discriminating players at different competitive levels.

Since the advantage of more advanced biological maturity extends across all levels of perceived quality, it is recommended to introduce regular assessment of biological maturity into selection procedures to ensure a fairer evaluation of players. Chronological age should be taken into account in all comparisons of motor abilities between maturation groups, because without it some of the differences may reflect the chronological difference itself rather than an actual difference caused by more advanced biological maturity. Since speed tests, particularly without the ball, show great discriminative power in differentiating bio-banded groups, the interpretation of sprint test results should be cautious, as they may favor more mature players and thereby mask other quality criteria. In contrast, agility tests do not show significant differences between maturity groups, making them less biased toward biological maturity, while at the same time proving to be significant predictors of quality, which makes them a more desirable tool for assessing player quality. Notably, when examined individually, both ball-oriented tests (sprint and slalom with the ball) showed a significant influence on perceived quality, which may underscore the importance of ball-handling abilities in the selection and identification process. The negative effect of chronological age on the estimated quality in the regression model suggests that, among players at a similar level of biological maturity, chronologically younger players may be perceived as more skilled. One possible explanation is that coaches may place greater value on the long-term developmental potential of younger players, as those who have reached a comparable level of biological maturity at an earlier chronological age may be viewed as having more time and opportunity for further improvement.

Two further limitations warrant mention. First, because estimated biological age and chronological age are correlated by construction, the coaching-related interpretation offered above should be read with some caution. The VIF diagnostics reported in Section 2.3 suggest that the negative chronological-age effect is not primarily a statistical artifact of collinearity; however, some residual overlap between the two predictors cannot be fully excluded, and the proposed coaching mechanism remains one plausible interpretation rather than an established one.

Second, the construct validity of the quality score (QS) merits discussion. QS combines the coach’s subjective rating with an adjustment for the player’s club competitive level, so the two components are not fully separable. Part of the association observed between QS and biological maturity could therefore, in principle, reflect club level rather than the coach’s technical or tactical judgment alone. As reported in Section 2.2.3, the distribution of bio-banded maturity groups did not differ significantly across the three competitive tiers used to adjust QS (χ^2^ = 6.77, *p* = 0.148), suggesting that this association is unlikely to be driven primarily by an uneven distribution of mature players across club levels. Nonetheless, because coach rating and club level are combined into a single score, and because inter-rater reliability could not be assessed, each player was rated by only one coach (Section 2.2.2); QS is best interpreted as a proxy for perceived quality within its competitive context, rather than a purely independent measure of technical and tactical ability.

Future research could address these limitations in several concrete ways. First, coach rating and club competitive level should be collected and analyzed as separate constructs, rather than combined into a single composite score, so that their relative contributions to perceived quality can be disentangled statistically (e.g., via multilevel models with club as a random effect). Second, inter-rater reliability should be formally established by having each player independently evaluated by at least two raters, with agreement quantified using intraclass correlation coefficients or weighted kappa. Third, given the correlation between chronological and estimated biological age inherent to equation-based maturity estimation, future studies may benefit from incorporating an independent, non-equation-based marker of somatic maturity (e.g., skeletal age from hand–wrist radiography, where ethically and practically feasible) to corroborate the Mirwald-based estimates used here. Finally, prospective longitudinal designs that track each player’s actual timing of peak height velocity, rather than classifying maturity status relative to the sample mean at a single time point, would allow future studies to more precisely characterize each player’s individual maturity-related trajectory.

## 5. Conclusions

This study confirmed the significant role of biological maturity in the perceived quality of young football players, with more advanced maturity associated with higher quality scores across all levels. Chronological age, when controlling for biological maturity, showed an opposite, negative effect. One possible explanation is that players who attain a given level of maturity at a younger chronological age may be perceived by coaches as more promising. These results may be due to the perception that these players have greater potential and more time for further development. Among motor abilities, agility emerged as a significant predictor of quality and appeared less biased toward biological maturity compared to speed tests. These findings highlight the importance of considering both biological and chronological age in selection procedures to ensure a fairer evaluation of players. These results suggest that talent identification based heavily on physical performance may favor biologically more mature players, since biological age was positively related to perceived quality, while speed appeared to reflect maturity to a greater extent than playing ability alone. Caution is therefore needed during selection, as players with comparable long-term potential may be overlooked simply because they are less physically developed at the time of assessment. Agility tasks proved to predict quality while remaining less influenced by maturity than speed tasks; among the ball-oriented tests, only the slalom with the ball showed comparable independence from maturity, whereas the sprint with the ball remained associated with biological maturity. Practitioners could combine assessments of ball-oriented tasks, motor performance and chronological and biological age during selection process. A holistic approach could improve talent identification and development as well as may promote a fairer evaluation of young players. Limitations of the study include the cross-sectional design, reliance on an equation for maturity assessment, and the construct validity and inter-rater reliability considerations regarding the quality score discussed above. Future research should examine these relationships longitudinally, using direct measures of maturity and including other potential predictors of quality, such as technical and tactical skills.

## Figures and Tables

**Table 1:** Differences in Estimated Biological Age Across Quality Score Groups and club ranking (raw and adjusted for chronological age).

Quality Score Group (QS)	*M* ± *SD*	*M* ± *SE* (ANCOVA)
Poorest (Q1); *n* = 22	13.680 ± 0.993 d, e	14.041 ± 0.127
Below average (Q2); *n* = 48	14.017 ± 0.991	14.176 ± 0.086
Average (Q3); *n* = 76	13.948 ± 1.075 d, e	13.950 ± 0.068 e
Good (Q4); *n* = 62	14.406 ± 1.088	14.195 ± 0.075
Best (Q5); *n* = 32	14.484 ± 1.084	14.401 ± 0.105

Note. *M* = mean; *SD* = standard deviation; *SE* = standard error of the adjusted mean; *n* = sample size; Quality score: 1 = poorest, 5 = best (coach-rated); d = significant difference from Q4; e = significant difference from Q5.

**Table 2:** Coefficients of the ordinal logistic regression (proportional odds model) predicting the quality category of U-15 players based on age indicators and motor performance tests with and without ball.

Predictor	*B*	*SE*	*t*	*p*	*OR*	*95% CI*
Estimated biological age (yrs.)	**0.91**	0.23	3.9	**<0.001**	2.47	1.57–3.90
Chronological age (yrs.)	**−0.82**	0.24	−3.48	**<0.001**	0.44	0.28–0.70
S20 (s)	0.34	0.24	1.39	0.165	1.40	0.87–2.25
S20 B (s)	**−0.84**	0.26	−3.19	**<0.001**	0.43	0.26–0.72
SLAL (s)	**−0.59**	0.19	−3.15	**0.002**	0.55	0.38–0.80
SLAL B (s)	**−0.61**	0.17	−3.67	**<0.001**	0.54	0.39–0.75

Note. *B* = unstandardized logit coefficient (predictors standardized to z-scores); *SE* = standard error; *t* = test statistic (B/SE); *p* = significance level; *OR* = odds ratio (=exp(B)); *95% CI* = confidence interval for OR, OR < 1 indicates that an increase in the predictor decreases the odds of belonging to a higher quality category.

**Table 3:** Differences in Quality Indicators, Morphological Characteristics, and Motor Performance Between Different Bio-Banded groups.

Variable	Less Mature (*n* = 93)	Average Mature (*n* = 56)	More Mature (*n* = 91)	*p*	*η* ^2^
	*M ± SD*	*M ± SD*	*M ± SD*		
***Quality***					SMALL
QS (1–5)	2.946 ± 1.107 c	3.089 ± 1.297	3.374 ± 1.092	0.040	
***Morphological and maturity characteristics***					
Chronological age (yrs.)	13.389 ± 0.553 b, c	14.136 ± 0.596 c	14.861 ± 0.490	<0.001	LARGE
BH (cm)	160.192 ± 6.914 b, c	171.627 ± 5.726 c	177.299 ± 6.263	<0.001	LARGE
BM (kg)	46.071 ± 6.554 b, c	58.136 ± 6.651 c	63.895 ± 7.278	<0.001	LARGE
BMI (kg/m^2^)	17.894 ± 1.764 b, c	19.700 ± 1.711 c	20.305 ± 1.899	<0.001	LARGE
APHV (yrs.)	14.165 ± 0.523 b, c	13.764 ± 0.547 c	13.368 ± 0.440	<0.001	LARGE
Estimated biological age (yrs.)	13.001 ± 0.448 b, c	14.147 ± 0.309 c	15.265 ± 0.460	<0.001	LARGE
***Speed and agility—without the ball***					
S20 (s)	3.454 ± 0.167 b, c	3.293 ± 0.217 c	3.219 ± 0.176	<0.001	LARGE
SLAL (s)	7.625 ± 0.538	7.699 ± 0.563	7.577 ± 0.452	0.376	TRIVIAL
***Speed and agility—with ball***					
S20 B (s)	3.634 ± 0.235 b, c	3.493 ± 0.232 c	3.412 ± 0.194	<0.001	LARGE
SLAL B (s)	10.997 ± 1.057	10.878 ± 1.004	10.828 ± 1.054	0.535	TRIVIAL
***Skill index (SI)***					
S20 SI	0.180 ± 0.145	0.200 ± 0.142	0.193 ± 0.119	0.647	TRIVIAL
SLAL SI	3.372 ± 0.830	3.179 ± 0.709	3.251 ± 0.833	0.331	TRIVIAL

Note. *M* = mean; *SD* = standard deviation; *n* = sample size; BH = body height; BM = body mass; APHV = age at peak height velocity; estimated biological age; QS = quality score (1 = poorest, 5 = best); S20 = 20-m sprint; SLAL = slalom test; S20 B = 20-m sprint with ball; SLAL B = slalom with ball; S20 SI = 20-m sprint skill index; SLAL SI = slalom skill index; *F* = F-ratio (df between, within); *η*^2^ = eta squared; b = significant difference from Average mature group; c = significant difference from More mature group.

**Table 4:** Differences in Morphological, Motor Performance, and Quality Indicators Between Different Bio-Banded groups, Adjusted for Chronological Age (ANCOVA).

Variable	Less Mature (*n* = 93)	Average Mature (*n* = 56)	More Mature (*n* = 91)	*p*	*η* ^2^
	*M ± SE*	*M ± SE*	*M ± SE*		
***Quality***					TRIVIAL
QS (1–5)	3.062 ± 0.119	3.087 ± 0.153	3.257 ± 0.120	0.699	
***Morphological and maturity characteristics***					
BH (cm)	160.451 ± 0.666 b, c	171.622 ± 0.858 c	177.037 ± 0.673	<0.001	LARGE
BM (kg)	46.330 ± 0.713 b, c	58.130 ± 0.918 c	63.633 ± 0.720	<0.001	LARGE
BMI (kg/m^2^)	17.944 ± 0.187 b, c	19.699 ± 0.242	20.254 ± 0.190	<0.001	MEDIUM
APHV (yrs.)	14.610 ± 0.039 b, c	13.755 ± 0.050 c	12.918 ± 0.039	<0.001	LARGE
Estimated biological age (yrs.)	13.279 ± 0.039 b, c	14.141 ± 0.050 c	14.985 ± 0.039	<0.001	LARGE
***Speed and agility—without ball***					
S20 (s)	3.382 ± 0.018 b, c	3.294 ± 0.023	3.292 ± 0.018	0.026	SMALL
SLAL (s)	7.426 ± 0.051 b, c	7.703 ± 0.066	7.777 ± 0.052	0.004	SMALL
***Speed and agility—with ball***					
S20 B (s)	3.557 ± 0.022	3.494 ± 0.028	3.490 ± 0.022	0.269	SMALL
SLAL B (s)	10.637 ± 0.105	10.885 ± 0.135	11.191 ± 0.106	0.060	SMALL
***Skill index (SI)***					
S20 SI	0.175 ± 0.014	0.200 ± 0.018	0.198 ± 0.014	0.614	TRIVIAL
SLAL SI	3.211 ± 0.083	3.182 ± 0.107	3.414 ± 0.084	0.307	TRIVIAL

Note. *M* = mean; *SE* = standard error of the adjusted mean; *n* = sample size; BH = body height; BM = body mass; APHV = age at peak height velocity; estimated biological age; QS = quality score (1 = poorest, 5 = best); S20 = 20-m sprint; SLAL = slalom test; S20 B = 20-m sprint with ball; SLAL B = slalom with ball; S20 SI = 20-m sprint skill index; SLAL SI = slalom skill index; *F* = F-ratio (df between, within); *η*^2^ = eta squared; b = significant difference from Average mature group; c = significant difference from More mature group.

## Data Availability

Data are available from the corresponding author upon reasonable request, subject to applicable ethical, legal, institutional, and confidentiality restrictions.

## Supplementary Materials

The following supporting information can be downloaded at: https://www.mdpi.com/article/10.3390/sports14080342/s1, Table S1: Sensitivity analysis: ordinal logistic regression coefficients using the maturity offset as a single predictor in place of the two separate age terms.
