# Supplementary material for: Do Biological Maturity and Motor Performance Influence Perceived Player Quality? Evidence from U-15 Soccer Players
Source: Sports (Basel). 2026 Aug 7;14(8):342. doi: 10.3390/sports14080342 (PMC13517613; doi:10.3390/sports14080342)
Supplement: Supplementary file 1 [file sports-14-00342-s001.zip › sports-4461018-supplementary.pdf]

Table S1. Sensitivity analysis: ordinal logistic regression coefficients using the maturity offset as a single predictor in place of the two separate age terms.

**Predictor**	*B*	*SE*	*t*	*p*	*OR*	*95%CI*
Maturity offset	**0.51**	0.13	3.90	**< 0.001**	1.66	1.29-2.15
S20 (s)	0.40	0.23	1.73	0.084	1.49	0.95-2.33
S20 B (s)	**-0.84**	0.26	-3.19	**0.001**	0.43	0.26-0.72
SLAL (s)	**-0.59**	0.19	-3.18	**0.001**	0.55	0.38-0.80
SLAL B (s)	**-0.60**	0.16	-3.64	**< 0.001**	0.55	0.40-0.76

Note. B = unstandardized logit coefficient (predictors standardized to z-scores); *SE* = standard error; *t* = test statistic (B/SE); *p* = significance level; OR = odds ratio (= exp(B)); 95% CI = confidence interval for OR. Model N = 240; AIC = 629.4 (original two-predictor age specification: AIC = 630.8).
